# Spontaneous Genomic Variation as a Survival Strategy of Nosocomial Staphylococcus haemolyticus

**DOI:** 10.1128/spectrum.02552-22

**Published:** 2023-03-06

**Authors:** Ons Bouchami, Miguel Machado, João André Carriço, José Melo-Cristino, Hermínia de Lencastre, Maria Miragaia

**Affiliations:** a Laboratory of Bacterial Evolution and Molecular Epidemiology, Instituto de Tecnologia Química e Biológica António Xavier, Universidade Nova de Lisboa (ITQB-NOVA), Oeiras, Portugal; b Laboratory of Molecular Genetics, Instituto de Tecnologia Química e Biológica António Xavier, Universidade Nova de Lisboa (ITQB-NOVA), Oeiras, Portugal; c Instituto de Microbiologia, Instituto de Medicina Molecular, Faculdade de Medicina, Universidade de Lisboa, Lisbon, Portugal; d Laboratory of Microbiology and Infectious Diseases, Rockefeller University, New York, USA; Riverside University Health System, Medical Center, University of California

**Keywords:** Staphylococcus haemolyticus, methicillin resistance, insertion sequences, subpopulation, deletion, diversity, evolution, beta-lactams, coagulase-negative staphylococci, staphylococci

## Abstract

Staphylococcus haemolyticus is one of the most important nosocomial human pathogens frequently isolated in bloodstream and medical device-related infections. However, its mechanisms of evolution and adaptation are still poorly explored. To characterize the strategies of genetic and phenotypic diversity in *S. haemolyticus*, we analyzed an invasive strain for genetic and phenotypic stability after serial passage *in vitro* in the absence and presence of beta-lactam antibiotics. We performed pulsed-field gel electrophoresis (PFGE) of the culture and analyzed five colonies at seven time points during stability assays for beta-lactam susceptibility, hemolysis, mannitol fermentation, and biofilm production. We compared their whole genomes and performed phylogenetic analysis based on core single-nucleotide polymorphisms (SNPs). We observed a high instability in the PFGE profiles at the different time points in the absence of antibiotic. Analysis of WGS data for individual colonies showed the occurrence of six large-scale genomic deletions within the *oriC* environ, smaller deletions in non-*oriC* environ regions, and nonsynonymous mutations in clinically relevant genes. The regions of deletion and point mutations included genes encoding amino acid and metal transporters, resistance to environmental stress and beta-lactams, virulence, mannitol fermentation, metabolic processes, and insertion sequence (IS) elements. Parallel variation was detected in clinically significant phenotypic traits such as mannitol fermentation, hemolysis, and biofilm formation. In the presence of oxacillin, PFGE profiles were overall stable over time and mainly corresponded to a single genomic variant. Our results suggest that *S. haemolyticus* populations are composed of subpopulations of genetic and phenotypic variants. The maintenance of subpopulations in different physiological states may be a strategy to adapt rapidly to stress situations imposed by the host, particularly in the hospital environment.

**IMPORTANCE** The introduction of medical devices and antibiotics into clinical practice have substantially improved patient quality of life and contributed to extended life expectancy. One of its most cumbersome consequences was the emergence of medical device-associated infections caused by multidrug-resistant and opportunistic bacteria such as Staphylococcus haemolyticus. However, the reason for this bacterium’s success is still elusive. We found that in the absence of environmental stresses, S. haemolyticus can spontaneously produce subpopulations of genomic and phenotypic variants with deletions/mutations in clinically relevant genes. However, when exposed to selective pressures, such as the presence of antibiotics, a single genomic variant will be recruited and become dominant. We suggest that the maintenance of these cell subpopulations in different physiological states is an extremely effective strategy to adapt to stresses imposed by the host or the infection environment and might contribute for *S. haemolyticus* survival and persistence in the hospital.

## INTRODUCTION

Staphylococcus haemolyticus is one of the most important nosocomial human pathogens frequently isolated in blood infections (including sepsis) related to implanted medical devices (1–4). It is easily distinguished from other coagulase-negative staphylococci by its multidrug-resistance pattern (5), formation of thick biofilms (6) and high phenotypic variation, and genome plasticity (7).

Its genetic diversity is believed to result from its large number of insertion sequence (IS) elements (7). As many as 82 of these IS elements were described in *S. haemolyticus* JCSC1435 genome, of which 60 were intact. These are many more than those found in *Staphylococcus epidermidis* ATCC 12228 (18 intact IS) and *Staphylococcus aureus* Mu50 (13 intact IS). The large amount of IS elements is believed to contribute to its genome plasticity through chromosomal rearrangements or insertions/deletions. However, the mechanisms of the evolution and adaptation of *S. haemolyticus* are still poorly understood.

Insertion sequences are transposable elements (less than 2.5 kb) that carry no genetic information except for transposases and short flanking terminal inverted repeat sequences (IR) (between 10 and 40 bp), which serve as recognition sites for the transposase. This enzyme usually excises the IS and inserts it elsewhere in the genome (conservative transposition), but occasionally the IS replicates during the transposition process (replicative transposition) (8, 9). By using mechanisms independent of large regions of DNA homology between the IS and target, these transposable elements are capable of repeated insertion at multiple sites within a genome. The impact of ISs in the overall genome architecture and gene expression can be very important, especially when they are present in multiple copies (10–12). These elements often cause gene inactivation (by direct integration into an open reading frame) and have strong polar effects, but they can also lead to the activation (by providing the gene with a potent promoter) or alteration of the expression of adjacent genes (13). By changing the content of the genome, IS elements may contribute to the innate ability of the bacterium to acquire drug resistance (14–16). Moreover, they can lead to complex chromosomal rearrangements that result in inversions or deletions, which can be very large and have impact on host adaptation (17).

The impact of IS transposition on *S. haemolyticus* chromosomal rearrangements have only been explored in the strain JCSC1435, which has 56 copies (40 intact) of IS*1272* and IS*Sha1*. In this strain, genomic rearrangements occurred preferentially near the origin of replication and implicated the deletion/inversion of large chromosomal fragments, which affected antibiotic resistance and sugar metabolism (7). However, it is not known how frequently this phenomenon occurs within the population, whether it is restricted to the *ori* region, whether it also occurs at the cell population level, which factors might induce it, and what are the consequences for bacterial fitness and survival. The *oriC* environ is a chromosomal region of staphylococci reported to be important for the evolution of staphylococcal species, and it is significantly larger in *S. haemolyticus* than in S. aureus and S. epidermidis. For example, this region integrates the staphylococcal cassette chromosome, conferring resistance to virtually all beta-lactams (18).

Another possible origin of genetic diversity in *S. haemolyticus* which may or may not be related to IS is the high recombination rate described based on examination of the sequence changes at MLST loci during clonal diversification (19). In particular, the per-allele and per-site recombination-to-mutation (r/m) rates reported for this species are 1:1 and 2.9:1, respectively (19).

The few studies available analyzing the population structure of nosocomial *S. haemolyticus* have shown that they are genetically diverse by pulsed-field gel electrophoresis (PFGE); however, they belong to two main clonal lineages, as concluded by multilocus sequence typing (MLST) (19, 20) and whole-genome sequencing analysis (21).

In spite of the extremely high number of ISs in *S. haemolyticus* and the high recombination rate the impact of transposition and recombination in genome architecture, population structure, and pathogenicity of *S. haemolyticus* was limitedly explored only. This study showed that chromosomal and phenotypic diversity in *S. haemolyticus* frequently occurs within a cell population, revealing a new mechanism of the evolution and adaptation of this species.

## RESULTS

### *S. haemolyticus* invasive strain has high genomic instability in the absence of environmental stress.

In our previous study (19), we observed high instability in SmaI PFGE macrorestriction patterns during serial passage *in vitro* in optimal growth conditions for the invasive *S. haemolyticus* strain HSM742. A culture originated from a single colony was the starting point of a stability assay which was performed over 34 days. The growth rate of HSM742 strain was 0.33 h^−1^, which corresponds to a duplication time of 2 h, indicating that after 34 days of serial growth *in vitro*, 408 generations had occurred. During this period, the SmaI PFGE patterns changed several times in two or more bands and on some occasions reverted to the original genotype. The PFGE patterns of *S*. *haemolyticus* strain HSM742 on days 4, 7, 10, 19, and 31 remained unchanged after passage (results shown in previous study [19]; Fig. S1) compared to day 0. However, the PFGE patterns obtained from days 13, 16, 22, 25, and 28 were distinguishable from those of the original (first) strain. We noticed that the banding patterns of strains isolated on days 13, 25, and 28 varied at three loci: one with a new band (approximate molecular weight = 358 Kb) and two with missing bands (approximate molecular weights = 432 and 74 Kb). The PFGE patterns of strains isolated in days 16 and 22 each lost one band (approximately 74 and 108 Kb, respectively) and gained an additional band (432 and 159 Kb, respectively).

These results suggest that in the absence of an environmental stress, there are diverse subpopulations of genomic variants in the same cell culture.

### Genomic variants of *S. haemolyticus* invasive strain have deletions within and outside the *oriC* environ.

**FIG 1:**
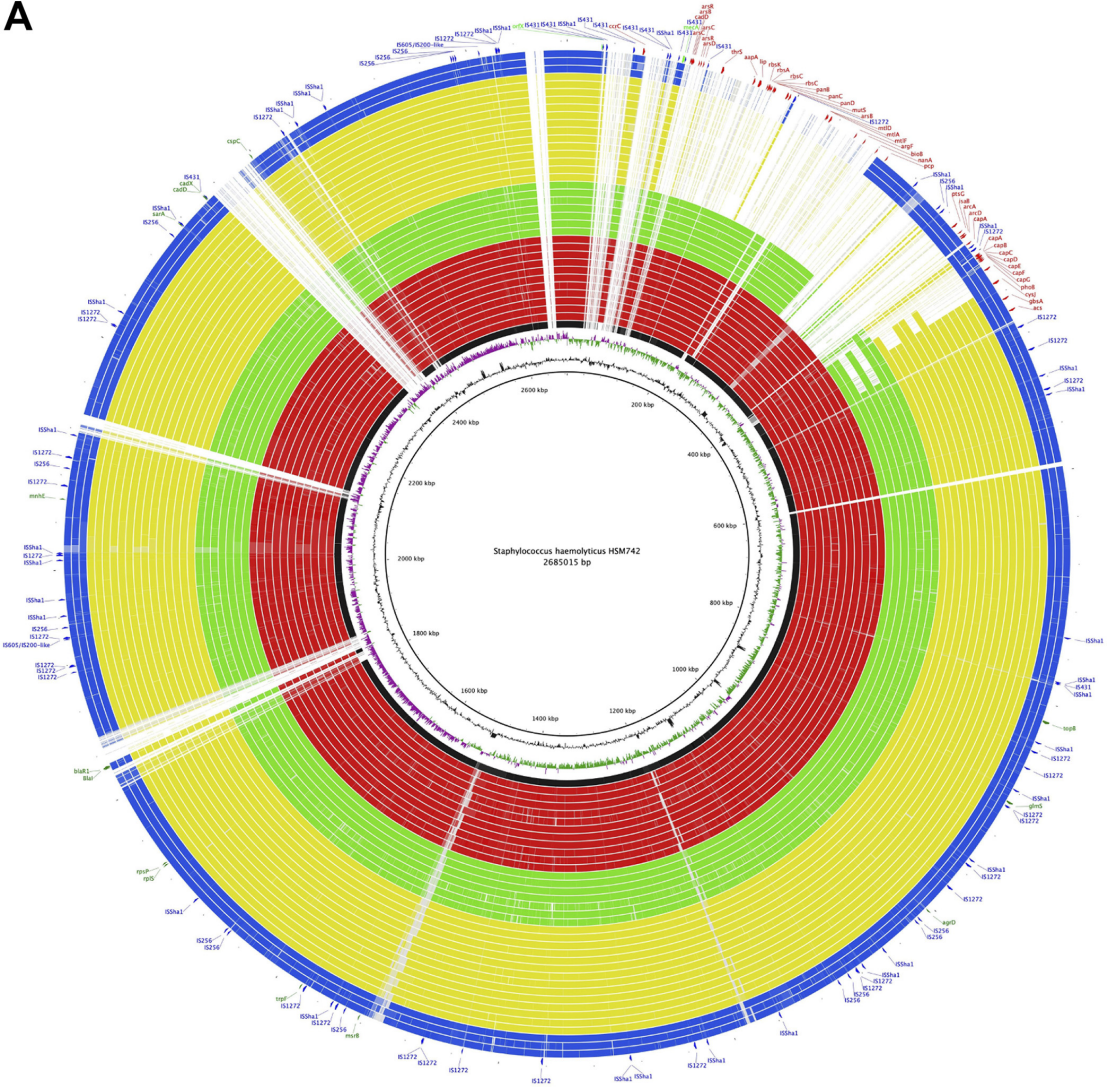

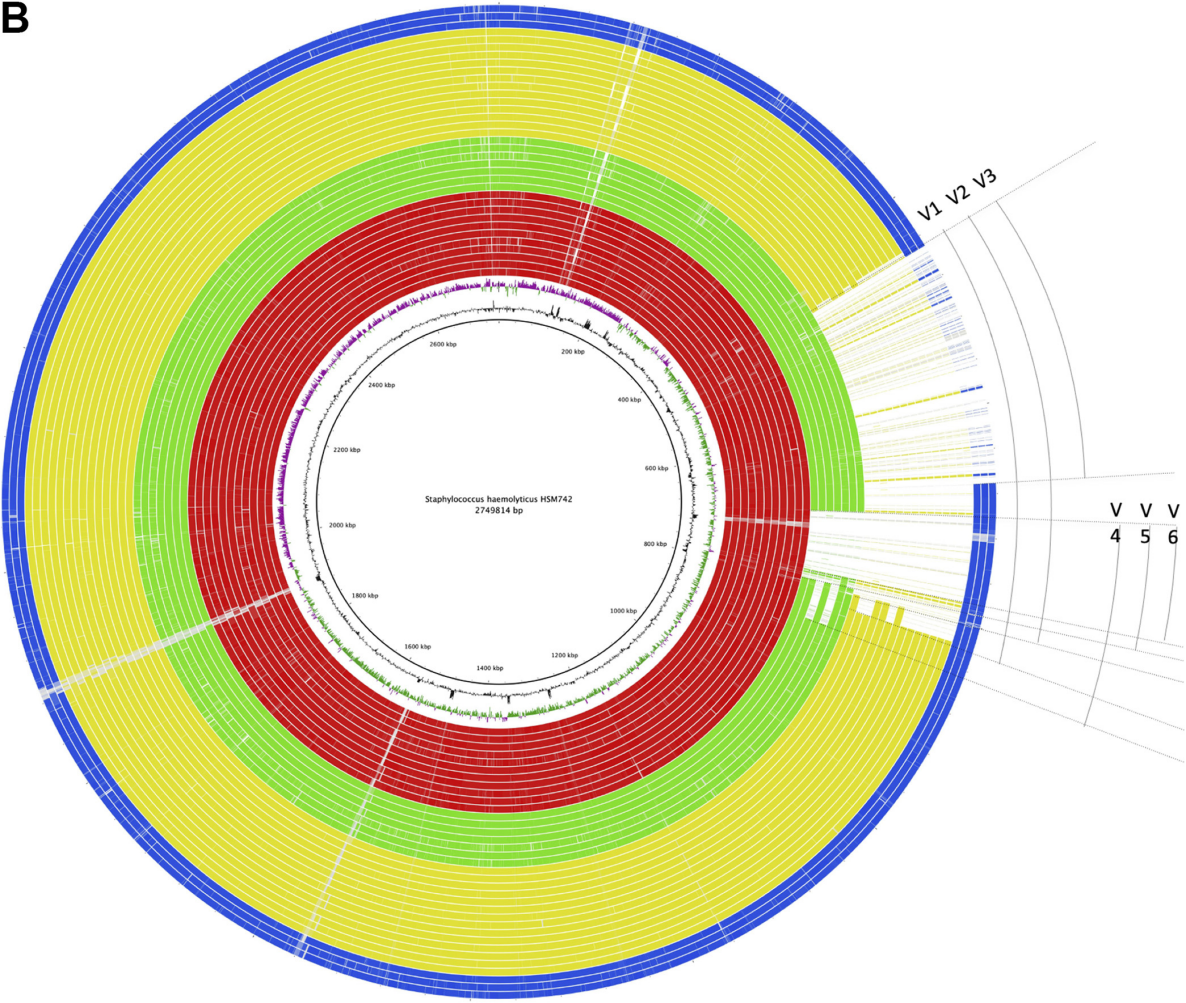
BLAST Ring Image Generator (BRIG) diagram showing homologous chromosome segments of Staphylococcus haemolyticus HSM742 strains with genomes of strains JCSC1435 or HSM742d0 as references. (A) BRIG circular diagram of the HSM742 chromosome showing (from inner to outer) homology (based on BLASTn+ analysis) of *S. haemolyticus* JCSC1435 reference genome to 35 HSM742 complete genomes (24 mannitol-negative and 11 mannitol-positive strains). Innermost circles represent the GC content (black) and GC skew (purple/green). Outer rings show shared identity (according to BLASTn) with individual HSM742 genomes and JCSC1435 genome. BLASTn matches between 70% and 100% nucleotide identity are colored from lightest to darkest shade, respectively. Matches with >70% identity, or JCSC1435 regions with no BLAST matches, appear as blank spaces in each ring. Colored circles from inside to outside as are follows: JCSC1435 (black); Sh29/312/L2 (black); d0C1-d0C5, d13C1 and -C2, d16C1 and -C2, d28C1, d34C1 (red); d13C3 (V5), d16C3 (V5), d16C5 (V4), d25C1 (V5), d25C2 (V4), d28C2 (V6), d34C4 (V5); d13C4 (V2) (green); d13C5 (V1), d16C4 (V1), d22C1-C2, d22C3 (V1), d22C4 (V2), d22C5 (V1), d25C3 to -C5 (V1), d28C3 to -C5 (V1) (yellow); d34C2 and -C3 (V3), and d34C5 (V3) (blue). Outer circle indicates location of the insertion sequences (blue labels and arcs) and regions of difference (deleted regions; red labels and arcs) not present in mannitol-negative strains, deleted genes outside the *oriC* region (green), and present genes (yellow). (B) BRIG circular diagram of the HSM742 chromosome showing (from inside to outside) homology based on BLASTn+ analysis of the closed genome of HSM742d0 to 35 HSM742 complete genomes (24 mannitol-negative and 11 mannitol-positive strains). Innermost circles represent GC content (black) and GC skew (purple/green). Outer rings show shared identity (according to BLASTn) with individual HSM742 genomes and HSM742d0 (sequenced by Nanopore and *de novo* assembled with Unicycler). BLASTn matches between 70% and 100% nucleotide identity are colored from lightest to darkest shade, respectively. Matches with >70% identity, or HSM742d0 regions with no BLAST matches, appear as blank spaces in each ring. Colored circles arranged from inside to outside are as follows: HSM742d0 (black); d0C1-d0C5, d13C1 and -C2, d16C1 and -C2, d28C1, d34C1 (red); d13C3 (V5), d16C3 (V5), d16C5 (V4), d25C1 (V5), d25C2 (V4), d28C2 (V6), d34C4 (V5); d13C4 (V2) (green); d13C5 (V1), d16C4 (V1), d22C1 and -C2, d22C3 (V1), d22C4 (V2), d22C5 (V1), d25C3 to -C5 (V1), d28C3 to -C5 (V1) (yellow); d34C2 and -C3 (V3), and d34C5 (V3) (blue). Images were prepared using BLAST Ring Image Generator (http://sourceforge.net/projects/brig).

To test this hypothesis and understand the mechanisms explaining the existence of genomic variant subpopulations in the absence of antibiotic, we selected the seven time points, at which we observed alterations in the PFGE patterns, to study in more detail (days 0, 13, 16, 22, 25, and 28). The cultures from each of these days were plated in rich medium and five colonies on each plate were randomly picked for DNA extraction and whole-genome analysis. Draft genomes of the 35 colonies were reconstructed by *de novo* assembly followed by alignment using Mauve (22) and the reference strain with a closed genome JCSC1435 (7). Draft genomes were then aligned and visualized using the BLAST Ring Image Generator (BRIG). A visual inspection of the circular alignment of the genomes of HSM742 (Fig. 1A) revealed a relatively high similarity of the draft genomes with the reference genome (90 to 100%), suggesting that almost all of the reference strain genome was covered by the Illumina sequencing performed for the 35 colonies. In addition, alignment of JCSC1435 with the closed genome obtained for HSM472 strain on day 0 using Nanopore showed that the two strains were highly homologous (92% identity) and had similar chromosomal structures, suggesting that JCSC1435 is an appropriate reference to use for alignment.

**FIG 2:**
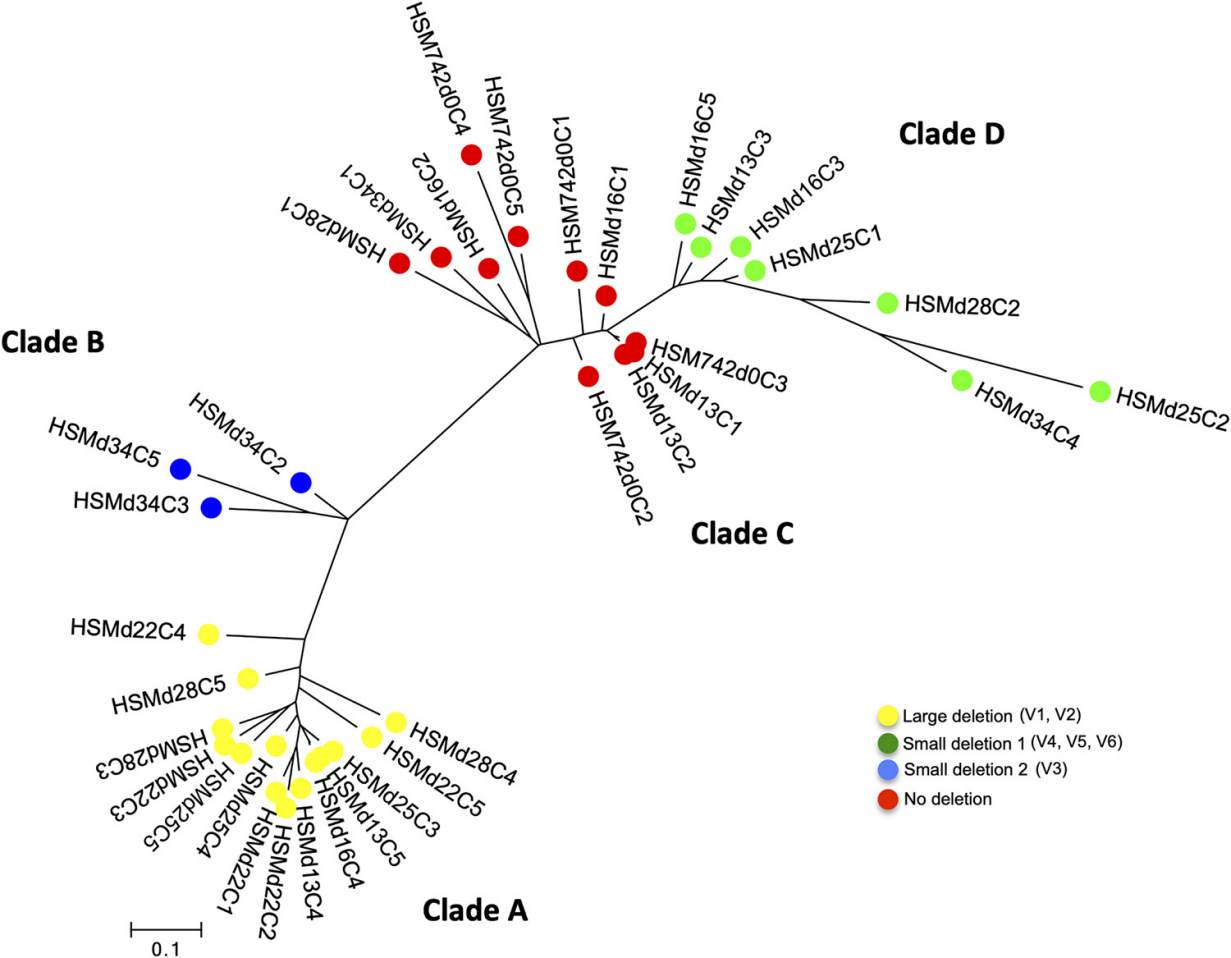
Tree constructed from the binary presence/absence of genes in the pangenome as defined by Roary in the genomes of 35 colonies obtained at different time points during a stability assay of *S. haemolyticus* strain HSM472, using the genome of JCSC1435 strain as the reference. Each node represents a strain (1 colony); nodes with identical color belong to the same cluster.

The genomes of the vast majority of colonies (obtained from up to 34 days of serial growth and >400 generations) were highly identical in their core nucleotide sequences, as shown by the small number of single-nucleotide polymorphisms (SNPs) found (12 to 295 SNPs, excluding mutators) when strains were compared by core SNP analysis (Fig. 2, Tables S1 and S2). However, six different structural genomic variants (V1 to V6) were observed when colonies’ *de novo* assembled contigs were aligned against the closed genomes of *S. haemolyticus* JCSC1435 and the HSM472-d0C1 (corresponding to a colony collected on day 1) using BRIG, or when their reads were mapped against the JCSC1435 strain (Fig. 1A and B). Genomic variants included deletions of different fragment sizes (313, 294, 179, 131, 82, and 74 Kb), all located in the *oriC* environ right to the origin of replication between nucleotides 36,000 and 349,000 bp. Five colonies did not suffer any deletion in the *oriC* compared to HSM742 d0C1.

To understand which genes were contained within these regions, we constructed a pangenome of the 35 colonies and recorded the presence/absence of genes using ROARY software. The largest deletion of 313 Kb accounted for the loss of as many as 310 genes (Table S3) (V1) compared to d0C1. This deletion event was observed in 10 colonies in the stability assays (d13C5, d16C4, d22C3, d22C5, d25C3 to -C5, and d28C3 to -C5) and occurred between MaoC domain-containing protein dehydratase (36Kb) and phosphoadenosine phosphosulfate reductase (CysH) (349 Kb). Another large fragment deletion, of a similar size (294 Kb) and spanning the same chromosomal region except for the last 23 bp, was also observed (V2) in four colonies (d13C4, d22C1 and -C2, and d22C4).

There were no insertion sequences near the extremities of these fragments; however, IS*1272*, IS*431,* and IS*Sha2* were part of the deletion fragment in all 14 colonies (Table S3). We also identified smaller deletions of 179 Kb (V3) within the same chromosomal region in three colonies (d34C2 to -C3 and d34C5), wherein 186 genes were lost in the *oriC* environ. The upstream deletion point coincided with those of variants V1 and V2, but the downstream deletion point in these colonies was at the end of the ABC transporter ATP-binding protein gene (215 Kb) instead.

The remaining three variants (V4, V5, and V6) corresponded to minor fragment deletion sizes (131, 82, and 74 Kb, respectively) and were detected in seven colonies (V4, d16C5 and d25C2; V5, d13C3, d16C3, d25C1, and d34C4; V6, d28C2). In these variants, 71, 78, and 123 genes, respectively, were deleted compared to d0C1, and IS*1272* was located in the upstream extremity of the deletion region in 5/7 isolates. These very small deletions occurred between the major facilitator superfamily (MFS) transporters, the chloramphenicol:cation symporter (located 244 Kb downstream from *oriC*), and a hypothetical protein (at 375 Kb, V4), the capsular polysaccharide biosynthesis protein Cap5G (at 326 Kb, V5), or a flavin mononucleotide-dependent NADH-azoreductase, AzoR protein (at 318 Kb, V6).

We identified yet another source of variation outside the *oriC* environ in different regions of the chromosome, wherein small deletions were also observed (Table S4). In the 35 colonies analyzed, 71 to 313 genes were deleted outside the *oriC*, which corresponds to 16% of the total number of genes initially present in d0C1 (predicted number = 1,893). We could not observe a direct correlation between the gene deletions occurring outside the *oriC* environ and those occurring within the *oriC* environ, suggesting that the two events are independent.

### Genomic variants of *S. haemolyticus* invasive strain are highly related.

To confirm the relatedness of the 35 colonies analyzed, we performed a SNP analysis of the draft genomes obtained for each strain. The percentage of the reference genome JCSC1435 which was covered by all isolates was 65.98%, implying that 1,771,593 positions from the reference were found in all analyzed genomes. A total of 5,551 qualified core SNPs without recombination events was used to construct SNPs distance matrices (see Supplemental File 1 and 2).

The great majority of 35 colonies differed between them by a small number of core SNPs (12 to 295 SNPs) (Supplemental File 1 and 2) (estimated medium short-term mutation rate: 3.7 × 10^−4^ substitutions per site per year) compared with the number of SNPs obtained when each colony was aligned to a completely distinct *S*. *haemolyticus* strain (JCSC1435, >5,300 SNPs). The only exceptions were d0C5 and d25C2, which were more distantly related to the remaining colonies (370 to 1,127 and 995 to 1,127 SNPs, respectively) and may correspond to mutator genotypes. A detailed analysis of the SNPs of d25C2 (V4) and d0C5 (no deletion) confirmed the existence of several nonsynonymous substitutions, compared to other colonies, in genes involved in DNA repair function which were previously associated with mutator phenotypes (23, 24). In the d25C2 colony, these included mutations in *mutL* (1 SNP difference in 32 variants and several SNPs in 2 variants), *recD* (1 to 4 SNPs in all variants), and *recN* (1 SNP in d0C4 only). Moreover, in the d0C5 colony, mutations in *mutL* (several SNPs in d34C1, -C3, and -C4), *recO* (several SNPs in d34C4 only), and *recU* (1 SNP in d28C2 only) were additionally found. *recD*, *recN*, and *recU* are known to be involved in genetic recombination and DNA repair in several bacteria, such as S. aureus, Escherichia coli, and Bacillus subtilis, and alterations in these genes may lead to accumulation of mutations in DNA and the inability of bacteria to recombine (25–30).

A tree based on gene presence/absence grouped the 35 *S. haemolyticus* strains into four clades (A to D) (Fig. 2). The distribution of the six genomic variants coincided exactly with the clustering distribution. In particular, we observed that all the colonies with large-scale deletions, including V1 and V2 variants, were grouped in the same genomic cluster A (1 to 52 SNPs); colonies with small deletions, including variant V3, was grouped in clade B (61 to 167 SNPs); and strains, including the variants V4 to V6, were grouped in clade D (1 to 144 SNPs). Strains without deletions were clustered together in clade C (2 to 193 SNPs).

We also noticed that each cluster of related genomes included colonies isolated on different days of the stability assay. For example, in cluster A, we found highly related colonies isolated on days 13, 16, 22, 25, and 28; while in cluster C, we found highly related colonies isolated on days 0, 13, 16, 28, and 34. On the other hand, we also observed that on day 0, all the colonies tested had no deletions and all belonged to clade C, while on day 13, three colonies were deletion variants belonging to clades A, B, and D. The proportion of the different genomic variants actually seems to vary over time. Although after just a few generations (day 0), the non-deleted genome appeared to predominate, by day 22 (275 generations) the large-deletion variants (V1 and V2) had prevailed. This change in proportion was further confirmed by testing a higher number of colonies (*n* = 20) from each day of culture, using the presence/absence of mannitol fermentation as a surrogate marker of the deletion event (Fig. S2; see results below). This is based on the fact that large deletions include the loss of the mannitol fermentation operon, as observed by annotation of the deleted fragments. Another observation that sustains this finding is the fact that in some PFGE patterns, faint bands were detected at certain time points which then became stronger at later time points, and vice versa (Fig. S1).

Altogether, these results suggest that deletion variants were either already present in the starting culture (d0) or emerged in early generations, although at a low prevalence. Deletion events should have occurred a limited number of times in the population and then been maintained in subsequent generations, evolving afterwards mainly through mutations. However, the proportions of the different deletion variants appear to have varied over time (Fig. 2).

### Estimation of the proportion of deletion variants in the population.

To establish the proportions of the variants at each time point, we took advantage of the fact that colony-variants suffering large deletions (V1, V2, and V3) had lost the mannitol operon and lacked the ability to ferment mannitol, while colonies without deletions (ND) and those with small deletions (V4, V5, and V6) were still able to metabolize this sugar. In fact, a total association could be observed for the five colonies tested at each time point regarding the type of variant, the presence/absence of the mannitol operon (*mtlA*, *mtlD*, and *mtlF*) and the corresponding ability to ferment mannitol. To increase the number of colonies analyzed and distinguish between these two types of variants at each time point, we plated the daily cultures analyzed in this study, picked 20 colonies, and tested them for mannitol fermentation on microtiter plates (Fig. S2). We found that the proportions of mannitol fermenters and non-fermenters varied over time. We discovered that among the 20 colonies tested, the proportions of mannitol-negative strains were as follows: day 0, 0/20 strains; day 13, 1/20; day 16, 1/20; day 22, 15/20; day 25, 18/20; day 28, 13/20; and day 34, 17/20.

### Biological functions lost through deletion events in genomic variants.

**FIG 3:**
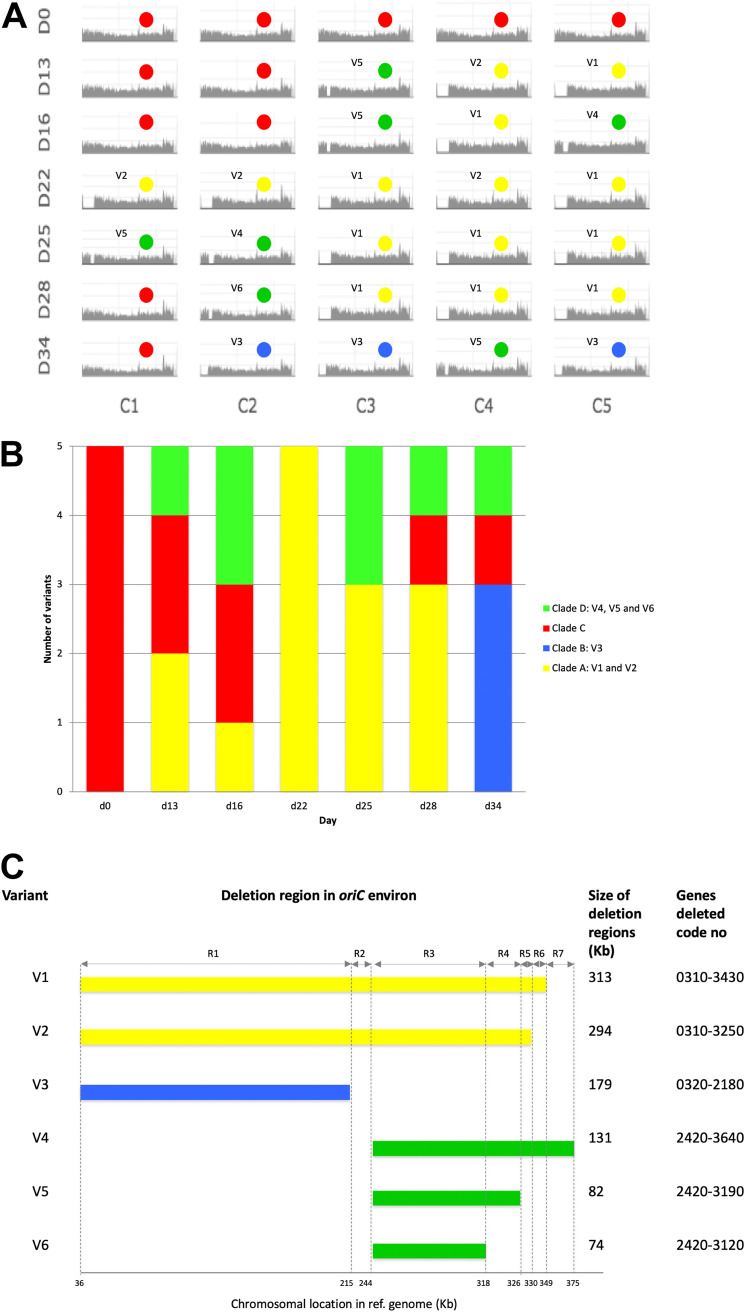
Distribution and chromosomal localization of deletion variants of strain HSM742 at seven time points during the stability assay. (A) Read mapping of strain HSM742 against the reference strain *S. haemolyticus* Sh29/312/L2. The *x* axes indicate the order of the nucleotides in the reference starting from the *oriC* region. The *y* axes indicate the coverage for each nucleotide position. D indicates days of passage *in vitro*; C indicates colonies. V1 to V6 indicate the deletion variants; colors represent the phylogenetic clades to which the colonies belong to according to SNP analysis: clade A, yellow; clade B, blue; clade C, red; clade D, green. (B) Distribution of deletion variants of strain HSM742 at seven time points in the stability assay. Colors indicate the clades to which each variant belong according to the phylogenetic tree constructed based on the presence/absence of accessory genes. (C) Chromosomal localization and distribution of deleted genomic regions in variants of strain HSM742. Color coding indicates the clades to which each variant belongs according to the presence/absence of accessory genes. Deletion sizes for each variant are given in kilobases (kb).

As summarized in Fig. 3 and Table S3, up to 310 genes were deleted from the largest deletion (V1), and all the other deletion variants suffered smaller deletions within this same region (V2, V3 to V6) or in a region 131-Kb upstream (V4). Among the deleted genes, a high proportion (*n* = 238) encoded hypothetical proteins. Functions could be attributed for only 72 genes. These included, for example, genes encoding metal transport systems and metal binding (arsenic, copper, chromium, and cadmium) (*arsB*, *copA*, *chrA*, and *cadD)*; transcriptional regulators (*arsR*, *asnC*, *cynR*, *lysR*); amino acid transporters (the d-serine/d-alanine/glycine transporter gene *aapA*); virulence-related genes (*isaB*, *lip*, *capABCDEFG*, *splE*, cell wall-anchored surface proteins); sugar transport and metabolism, namely, for mannitol (*mtlA*, *mtlD*, and *mtlF*), glucose/maltose/*N*-acetylglucosamine (*ptsG*), and ribose (*rbsABCK*); carbohydrate metabolism (*nanA*); mismatch repair (*mutS*); and restriction/modification systems (modification methylase DpnIIA: *DpnM*; NgoFVII family restriction endonuclease).

Among the 35 colonies, besides the large deletion in the *oriC* environ, we observed the deletion of up to 122 additional genes outside this region. A total of 276 genes encoding hypothetical proteins were also detected. The 122 genes with known functions included genes encoding or involved in insertion sequences, including IS*1272*, IS*431*, and IS*Sau3*; phages (prophages); metal and peptide transport (*tagH*, *potB*, *oppD*, *fmt*, *mnhE*, *vraG*, *copA*); metal binding (*csoR*, *sprT*); translation machinery (*rplU*, *rpml*, transfer RNAs); DNA replication (*dnaE*, *repC*); virulence, growth, and survival (T-box, *rli60*, and *putP*); methicillin and penicillin resistance (*blaR1*, *blaI*); quorum sensing and regulation of virulence (*agrC*); biofilm production (*traP*, *fnb*, *cadX*, *cadD*, *rli60*); response to osmotic and oxidative stress, acidic pH (ncRNA RsaA, RsaH), and cold shock (ncRNA RsaD); cell viability (*sipB*); and hemolysis (*agr*C).

The different deletion variants lost different functions compared to the genomes of non-deletion colonies (d0C1). For example, V3 colonies lost the ribose transporter and V4, V5, and V6 colonies lost the immunodominant antigen B (*isaB*). However, the maintenance of several different deletion variants in the same population (Fig. 3A and B and Table S3) guarantees that the entire gene pool is almost always present in the population.

### Phenotypic variation was observed during deletion events in genomic variants.

To evaluate the impact of the genomic deletion events in clinically relevant phenotypes, the same 35 colonies used to extract DNA for WGS were used to test for mannitol fermentation, oxacillin and cefoxitin resistance, biofilm formation, and hemolysis.

Different oxacillin and cefoxitin MICs were found between colonies, ranging from 32 to 256 μg/mL and 12 to >256 μg/mL, respectively. Additionally, we observed the existence of both mannitol fermenters and non-fermenters, hemolytic and non-hemolytic, and biofilm producers and non-producers among the 35 colonies. Moreover, difference in phenotypes were observed between colonies isolated from the same time point; namely, in terms of cefoxitin MICs, mannitol fermentation, hemolysis, and biofilm production (Table 1). This is the case for colonies collected on days 13, 16, 25, 28, and 34, which yielded both mannitol-positive and mannitol-negative results. On the other hand, the same phenotypes were found on different days of growth. For example, a cefoxitin MIC of 64 μg/mL was observed for 10 colonies collected on different days, including d13C3, d13C4, d16C4, d16C5, d25C4, d25C5, d28C3 to -C5, and d34C4.

**TABLE 1:**
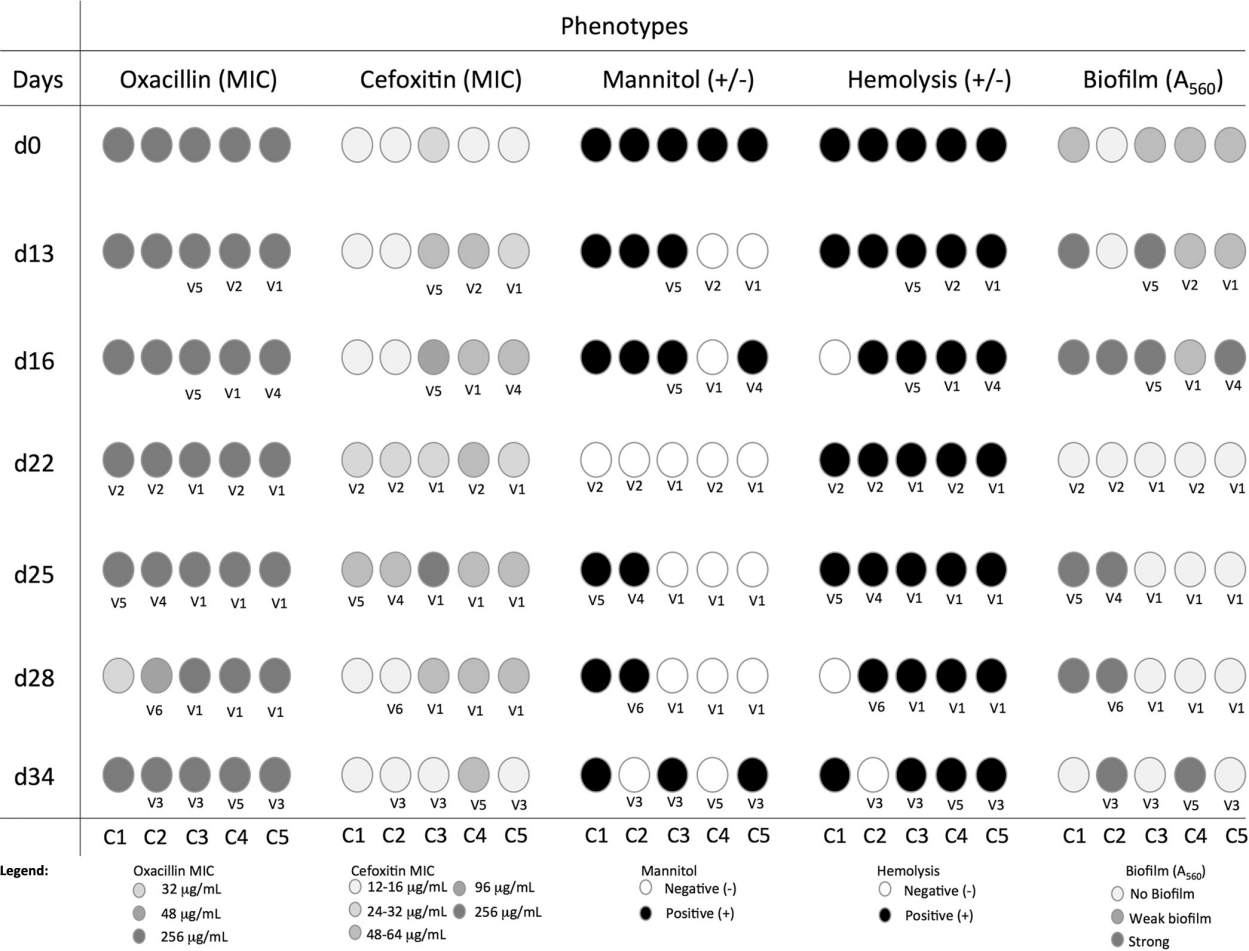
Colony-to-colony phenotypic variation of Staphylococcus haemolyticus strain HSM72 after 34 days of serial growth *in vitro*; alterations in phenotypes are shown in gray scale

### Establishment of associations between phenotypic and genotypic changes.

To identify potential links between phenotypic changes observed and the deletion events occurring in the *oriC* environ, described for the different colonies, we used a targeted approach in which we compared phenotypes obtained for each feature tested with the type of deletion variant(s) found for each colony (V1 to V6), searching for genes potentially associated with the phenotype within the deletion region. Additionally, to determine whether genes deleted in the region outside the *oriC* environ could be implicated in the emergence of phenotypic variants, we performed an untargeted association analysis. For this purpose, we constructed a pangenome of the 35 colonies using ROARY software and analyzed the presence/absence of genes. We also searched for the occurrence of nonsynonymous SNPs in genes potentially associated with the phenotypes tested.

All deletion variants except V3 and V6 were associated with an increase in the cefoxitin MIC compared to the colonies without deletions. These results suggest that the genes included in the region which is deleted in V1, V2, V4, and V5 but present in V3, V6, and non-deletion colonies (regions R2 and R4 in Fig. 3C), should be important for this change in phenotype. Of all the genes contained within regions R2 and R4, those whose functions have been previously associated with beta-lactam resistance include genes (*opuD*) encoding transport systems for osmolytes, such as choline/carnithine/betaine, which contribute to cytoplasm osmolarity. Several studies have shown that S. aureus strains exposed to cell wall inhibitors, such as beta-lactams, caused the upregulation of *opuD*, suggesting that the two systems are interconnected (31–33). *opuD* was also previously described to be upregulated and associated with increased beta-lactam susceptibility (decreased MIC) in *femAB* mutants of S. aureus and were found to be upregulated in a methicillin-resistant S. aureus (MRSA) heterogenous phenotype compared to a homogenous phenotype (34–36). In the presence of cell wall biosynthesis inhibitors such as beta-lactams, the peptidoglycan becomes fragile, and the cell becomes vulnerable to the high osmolarity of the bacterial cytoplasm (37). The induction of the osmoprotectant *opuD* may provide additional resistance to cell lysis and contribute to bacterial survival in the presence of antibiotics.

We also observed that variant V6 had an oxacillin MIC of 48 μg/mL, while all other deletion variants and the non-deletion colony had a MIC of >256 μg/mL; however, in this case, no region of deletion was specific to V6 only, and no genes were only present in this variant, as determined from the presence/absence analysis. An analysis of SNPs of the V6 variant (d28C2) showed mutations in *recU* (1-SNP difference in all variants; nonsynonymous substitution) and *femB* (1-SNP difference in d0C5 and d25C2 [V4]; nonsynonymous substitution) suggesting that this phenotype alteration could be associated with mutations in these genes, which have previously been reported to be involved in oxacillin resistance (Table 2) (38–41).

**TABLE 2:** Deletion regions and genetic determinants putatively involved in phenotype alteration in Staphylococcus haemolyticus HSM72 strain variants

Deletion region	Phenotype altered	Variant(s)	Genes (*n*)	Genes with attributed function putatively involved in phenotype alteration	Function	Genetic event(s)	Reference(s)
R1	Mannitol fermentation	V1, V2, V3	188	*mlt* operon	Oxidoreductase, mannitol-1-phosphate 5-dehydrogenase activity, phosphotransferase, mannitol metabolism pathway	Deletion	7
R2	Cefoxitin resistance	V1, V2	24	Oligopeptide ABC superfamily ATP binding cassette transporter binding protein	ATPase activity, ATP binding, transport, beta-lactam resistance	Deletion	46, 43, 44, 45
	Biofilm production				Biofilm	Mutations; *cadD*, *arsC*	47, 48
R3	Oxacillin resistance	V6	-	-	Beta-lactam resistance	Mutations; *recU*, *femB*	38, 40, 41, 39
R4	Biofilm production	V1, V2, V4, V5	46	Capsule polysaccharide	Pathway capsule polysaccharide biosynthesis, biofilm	Deletion	7
	Cefoxitin resistance			Choline/carnitine/betaine transporter	Transmembrane transporter activity, beta-lactam resistance	Deletion	46, 43
				Glycine betaine aldehyde dehydrogenase	Oxidoreductase, betaine-aldehyde dehydrogenase activity, beta-lactam resistance		
R5	-	V1, V2, V4	-	-	-	-	-

There was also an almost perfect correlation between the loss of mannitol fermentation ability and the deletion variants V1, V2, and V3. Although the deletion of the R1 region, which is common to these three variants, contains as many as 188 genes, the loss of mannitol fermentation ability is most likely explained by the deletion of the *mlt* operon contained within this region.

Regarding biofilm formation, the association between phenotype and genotype appears to be more complex. While all of the V1 and V2 colonies showed decreased biofilm formation compared to the non-deletion colonies, biofilm production was increased in the V4, V5, and V6 colonies, and V3 showed variable results. The genes associated with this decrease in biofilm formation may lie within the deletion region that is specific to V1 and V2 only (R2 region in Table 2 and Fig. 3C). Among the few annotated genes in this region, those encoding oligopeptide transport were previously found to be upregulated in biofilm compared to planktonic growth in S. aureus (35, 42) and may be also responsible for the decrease in biofilm formation when deleted in variants V1 and V2. The genes associated with increased biofilm formation should be related to a deletion region which was common and specific to V4, V5, and V6, or to genes which are specifically found in these variants and not in V1, V2, or the non-deletion colonies; however, according to the chromosomal deletion locations, this is impossible. It is thus conceivable that the strong-biofilm phenotype may be due to mutations in chromosome regions other than the *oriC* environ or to new regulatory mechanisms induced by the deletion event. Analysis of SNPs showed mutations in *cadD* and *arsC* (nonsynonymous substitutions) in strong-biofilm producers compared to weak-biofilm producers and non-producers, suggesting that this phenotype alteration may alternatively be due to mutations in these genes, which have been previously shown to be involved in biofilm formation (43–46).

Regarding hemolysis, no clear alteration in phenotype was observed, and the small variations in the hemolysis halo observed were not correlated with any particular deletion variant, gene, or mutation.

### In the presence of oxacillin, *S. haemolyticus* genome was stable.

The repetition of the stability assay from the original *S. haemolyticus* culture, under the exact same conditions but in the presence of oxacillin, showed results completely different from those obtained in the absence of an environmental stress. In particular, we found changes in PFGE patterns only on day 22. In this case, the strain on day 22 lost one band of approximately 159 Kb and gained one 108-Kb band. Additionally, the PFGE profile of the parental strain d0 in the presence of oxacillin was different from that in the absence of oxacillin. Interestingly, however, this PFGE pattern was similar to one of the PFGE pattern variants found in the stability assay performed in the absence of antibiotic (d22; Fig. S1).

To understand the similarities between the genomes of colonies grown in the presence and absence of the antibiotic in more detail, we sequenced the genomes of two colonies from the day 16 culture with oxacillin and one colony from day 22 without the antibiotic. In spite of having similar PFGE SmaI patterns, comparison of the genomes of these colonies showed that while d22 colony in the absence of oxacillin belonged to variant V4, the d16 colonies grown in the presence of antibiotic belonged to a new variant, V7 (Fig. S3). The V4 and V7 colonies differed in the presence/absence of 15 genes (*ermA*, *ydhk*, *pycA*, *copA*, *copB*, *blaZ*, *betA*, *acsA2*, *mutS*, *trpF*, *pepA1*, *isaA*, *cysl*, *glpD*, and *lagD*), one of which is directly involved in beta-lactam resistance (*blaZ*), as well as 4 transposases (IS*1181*, IS*Sau3*, IS*431*, IS*1381A*) and 29 genes with an unknown function. This corresponded to several deletions occurring mainly in the *oriC* environ.

Overall, we observed that in spite of the genomic diversity observed in the absence of an environmental stress, in the presence of sub-inhibitory concentrations of oxacillin, a single genomic variant became dominant.

## DISCUSSION

In this study, we have gained a deeper understanding of the mechanisms of chromosomal and phenotypic diversity in *S. haemolyticus* within a cell population by analyzing a nosocomial strain belonging to the most prevalent *S. haemolyticus* clonal type. This strain was analyzed for genetic and phenotypic stability after serial passages *in vitro* in the presence and absence of a physiological stress, using whole-genome sequencing, phylogenetic, and pan-genome analysis.

In our previous study (19), we found that the SmaI PFGE macrorestriction patterns of the invasive *S. haemolyticus* strain HSM742 were highly unstable during serial growth *in vitro* under optimal growth conditions. In this study, by sequencing five individual colonies at seven time points (*n* = 35), we found that the variability generated during serial growth in the absence of antibiotic was due to the existence of subpopulations of genomic variants derived from the same ancestral strain. This was supported by the low number of SNPs found when the core genomes of the 35 colonies was compared. Although the genomes of all colonies were highly related, we observed six different structural genomic variants (V1 to V6) which included deletions of different fragment sizes, all located in the *oriC* environ right to the origin of replication. This chromosomal region has been previously shown to be highly variable (7). Moreover, colony genomes suffered small deletions and nonsynonymous mutations in genes located in chromosome regions outside the *oriC* region. The deletions observed were mostly associated with insertion sequences which were either within the deletion fragment or in its extremity, suggesting that they may be involved in the deletion process. However, recombination events, previously shown to be frequent in *S. haemolyticus* (19), and mutation events may contribute to this diversity.

When a gene presence/absence tree was constructed for the 35 colonies, we found that each of the six deletion variants was grouped in a specific cluster of the tree, which was different from the cluster containing colonies without deletions. Each cluster included colonies isolated on different days of the stability assay and the same variants were detected at different time points. The only exception was cluster B, which only included colonies collected on day 34. This may be explained by the fact that V3, identified in cluster B, was predominant on day 34, but was present at a very low prevalence at other time points, and thus could not be detected by our approach, which examined a very small part of the cell population (*n* = 5 colonies). Alternatively, variants from this cluster may have been created on day 34 only. Additionally, the proportions of the different genomic variants varied over time. In conclusion, these results suggest that deletion variants were created in the population a limited number of times early in serial growth, and then maintained in subsequent generations, with their proportions changing over time. However, when serial growth was examined in the presence of sub-inhibitory concentrations of oxacillin, a specific variant, V7, was selected, as suggested by analysis of the PFGE patterns during serial growth under these conditions and confirmed by whole-genome sequencing. These results point toward the existence of subpopulations of variants as a survival strategy to counteract stress, wherein the most adapted variant will be the one which will be selected or created *de novo* and ultimately prevail.

In these variants, an impressive 71 to 310 genes in the *oriC* region were deleted compared to a colony from the starting culture (d0C1). The deleted genes included a plethora of different functions, namely, those encoding carbohydrate, sugar, metal, and amino acid transport; metabolic systems and metal binding; transcriptional regulators; virulence-related genes; mismatch repair; and restriction/modification systems. We also observed the deletion of additional genes outside the *oriC* region, such as insertion sequences (transposases) and phages (prophages); peptide transport; translation machinery; DNA replication; virulence, growth, and survival; methicillin and penicillin resistance; quorum sensing; biofilm production; response to osmotic, oxidative, and acidic stress and cold shock; and cell viability and hemolysis. Furthermore, when strains were compared by SNP analysis, nonsynonymous mutations in relevant genes were also detected. Moreover, we were able to demonstrate that the gene deletions and mutations detected were frequently paralleled by changes in clinically relevant phenotypes such as biofilm formation, beta-lactam resistance, and mannitol fermentation.

Altogether, deletions in and outside the *oriC* region represent nearly one-quarter of the *S. haemolyticus* chromosome and may constitute a mechanism of genome reduction by which bacteria would gain fitness (45, 47). On the other hand, it may be a specialization mechanism for *S. haemolyticus* to survive in a specific niche, as seen previously for other bacterial species (47–49). Although the different deletion variants lost distinctive functions, the maintenance of several different deletion variants in the same cell population, as observed in our study, guarantees that the entire gene library is present in the population at all time points. The existence of such a genetic diversity ensures that the most adapted variant will emerge from the population when it is faced with a new environmental challenge.

Although the study described here was entirely performed *in vitro*, a recent study by Both et al. (50) detected the presence of S. epidermidis deletion variants during prosthetic joint infections, suggesting that the mechanism described here for *S. haemolyticus* may actually occur *in vivo* and be a common strategy for coagulase-negative staphylococci to circumvent host immune defenses and stresses imposed by the hospital environment.

Overall, our results suggest that *S. haemolyticus* populations are composed of subpopulations of genetic variants which may affect their growth, gene expression level, stress resistance, specific metabolic processes, and virulence. The high genetic and phenotypic variability observed in the most epidemic *S. haemolyticus* clonal type appears to be the result of IS-dependent and IS-independent events such as recombination and mutation. The maintenance of subpopulations of cells in different physiological states may be a strategy to adapt rapidly to environmental stresses imposed by the host or hospital environment.

## MATERIALS AND METHODS

### Ethical statement.

Individual ethical approval and informed consent was not required for this study because the *S*. *haemolyticus* isolate was obtained as part of routine clinical diagnostic testing and was analyzed anonymously. Moreover, in this study, we analyzed the bacterial isolates, not the human subjects. The collection of this sample was in accordance with the European Parliament and Council decision for the epidemiological surveillance and control of communicable diseases in the European community (see https://eur-lex.europa.eu/LexUriServ/LexUriServ.do?uri=CONSLEG:2000D0096:20120905:EN:PDF).

### Bacterial strains.

The *S. haemolyticus* HSM742 strain used in this work was isolated from the blood culture of a 56-year-old male patient at a hospital in Portugal in 2010. This strain belonged to the most epidemic (most frequent and widely disseminated) *S. haemolyticus* clonal type (ST1, CC29) (19). Strains *S. haemolyticus* JCSC1435 and *S. haemolyticus* Sh29/312/L2 were used as references for WGS, the MRSA strain WIS was used as a control for stability assays, and S. aureus ATCC 29213 from the American Type Culture Collection (ATCC) was used as a control for antimicrobial susceptibility testing.

### Assessment of genomic stability *in vitro*.

*S. haemolyticus* strain HSM742 was subjected to serial passages on tryptic soy broth (TSB). A single colony was transferred to TSB (Difco Laboratories, Detroit, MI, USA) and grown for 24 h at 37°C. Cultures were daily transferred to fresh liquid medium (1:100 dilution) for 34 days (19). To assess genomic stability, the same set of experiments was carried out under antibiotic pressure using TSB supplemented with subinhibitory concentrations of oxacillin (MIC = 1/4) (Oxoid Limited, Basingstoke, United Kingdom). The MRSA isolate WIS was used as an internal control for the stability assay. Serially grown populations were characterized by PFGE (51).

### Evaluation of phenotypic stability *in vitro*.

Serially grown populations or colonies were tested for oxacillin and cefoxitin MICs, hemolysis halo, mannitol fermentation, and biofilm production.

Oxacillin and cefoxitin MICs were determined using E-tests (AB bioMérieux, Solna, Sweden) according to CLSI recommendations (52). Hemolysis was tested by spotting 5 μL drops of an overnight bacterial culture on the surface of blood agar plates for 48 h at 37°C (53). Mannitol fermentation was tested by inoculating isolates onto mannitol salt agar (Becton, Dickinson and Company, Le Pont de Claix, France) followed by incubation for 24 to 48 h at 37°C. Biofilm formation was detected using the microtiter plate assay method (6, 54).

### Assessment of cell population variability.

To assess variability within the same cell population, five colonies at seven time points during stability assays (*n* = 35 colonies) were analyzed for phenotypic features and whole-genome sequencing (WGS).

Serial dilutions of cultures corresponding to days 0, 13, 16, 22, 25, 28, and 34 were spread on the surface of tryptic soy agar and incubated overnight at 37°C. Five colonies (~17% to 100% of the population) were selected randomly from plates of the ancestral strain HSM742 (day 0) and from subsequent generations (days 13, 16, 22, 25, 28, and 34). Half of each colony was used to perform the phenotypic assays (oxacillin and cefoxitin resistance, hemolysis, mannitol fermentation ability, and biofilm production) and the other half was used for DNA extraction for whole-genome sequencing (WGS).

### Whole-genome sequencing and *de novo* assembly.

Genomic DNA was isolated from half of each colony using the Qiagen DNeasy blood and tissue kit (Qiagen, Limburg, The Netherlands) and sequenced using the Illumina MiSeq system. Libraries for genome sequencing were constructed using the Nextera XT DNA Sample Preparation kit (Illumina, San Diego, CA) and sequenced using 150 bp pair-end reads with an estimated coverage of 100×. After trimming, the reads were *de novo* assembled into contigs using the CLC Genomics Workbench 9.0 (Qiagen, Hilden, Germany) analysis package with default parameters.

Additionally, pure and high-molecular weight DNA was extracted from an overnight culture of d0C1 using a standard phenol-chloroform extraction protocol (55) and sequenced on a SpotON flow cell vR9.4.1 using Oxford Nanopore rapid barcoding protocol (Oxford Nanopore Technologies [ONT], Oxford, United Kingdom) MinION. Hybrid assembly using ONT data was done using Unicycler (56).

### Comparative genomic analysis.

All resulting contigs from Illumina sequencing were ordered using the closed genome of *S. haemolyticus* JCSC1435 (NCBI accession no. AP006716) as a reference with Mauve (http://darlinglab.org/mauve/mauve.html) (22). Automated annotation was performed using RAST (http://rast.nmpdr.org/) and Prokka (https://github.com/tseemann/prokka) software (57, 58) with the default settings. Genomic comparisons were performed using a combination of Mauve (22) and Artemis (http://www.sanger.ac.uk/science/tools/artemis-comparison-tool-act) (59). All genomes were visualized with BRIG (http://brig.sourceforge.net/) (60) using Blast with 70% and 90% as the lower and upper nucleotide identity thresholds, respectively, using both *S. haemolyticus* JCSC1435 and HSM742-d0C1 closed genomes as references. To examine the presence/absence of genes in all colonies, a pangenome was constructed using the Roary pipeline v3.12 (https://sanger-pathogens.github.io/Roary/) (61) with the default settings. A tree based on the binary presence/absence of accessory genes in the pangenome, as defined by Roary, was constructed using MEGA 7 software (62).

### Core-genome SNPs analysis.

SNPs were identified separately within each strain using the CSI Phylogeny v1.4 (https://cge.cbs.dtu.dk/services/CSIPhylogeny [63]) pipeline, available from the Centre of Genomic Epidemiology of the Technical University of Denmark. Mapping of the *de novo* assembled contigs against the JCSC1435 reference genome (GenBank accession no. AP006716) (7) was carried out using BWA v0.7.2 (64). Single-nucleotide polymorphisms were identified based on the mpileup files generated by SAMTools v0.1.18 (65). The criteria used for calling SNPs were as follows: minimum relative depth at SNP positions of 10%, minimum Z-score of 1.96, minimum SNP quality of 30, and minimum read-mapping quality of 25. The minimum distance between SNPs was disabled and all indels were excluded. An alignment of the SNPs was then created by concatenating the SNPs based on their position on the reference genome.

Gubbins software with default settings was used to detect the recombinant regions based on the SNP density (66). First, the polymorphic sites resulting from recombination events were detected and filtered out and the phylogeny was reconstructed using RAxML. The filtered SNP output was transformed into an SNP distance matrix using snp-dists v0.62 (https://github.com/tseemann/snp-dists). The short-term mutation rate was estimated considering the number of core SNPs between a colony on day 0 and a colony collected on day 34 that did not suffer deletions.

### Data availability.

All relevant data are provided within the manuscript and its Supplemental Material files.

The whole-genome raw sequencing reads data of the 35 isolates analyzed in this study have been submitted to the Sequence Read Archives under accession no. PRJNA836617.
