# Supplementary material for: Spontaneous Genomic Variation as a Survival Strategy of Nosocomial Staphylococcus haemolyticus
Source: Microbiol Spectr. 2023 Mar 6;11(2):e02552-22. doi: 10.1128/spectrum.02552-22 (PMC10100732; doi:10.1128/spectrum.02552-22)
Supplement: Supplemental file 5 — Figures S1 to S3. Download spectrum.02552-22-s0001.pdf, PDF file, 12.1 MB [file spectrum.02552-22-s0001.pdf]

Figure S1

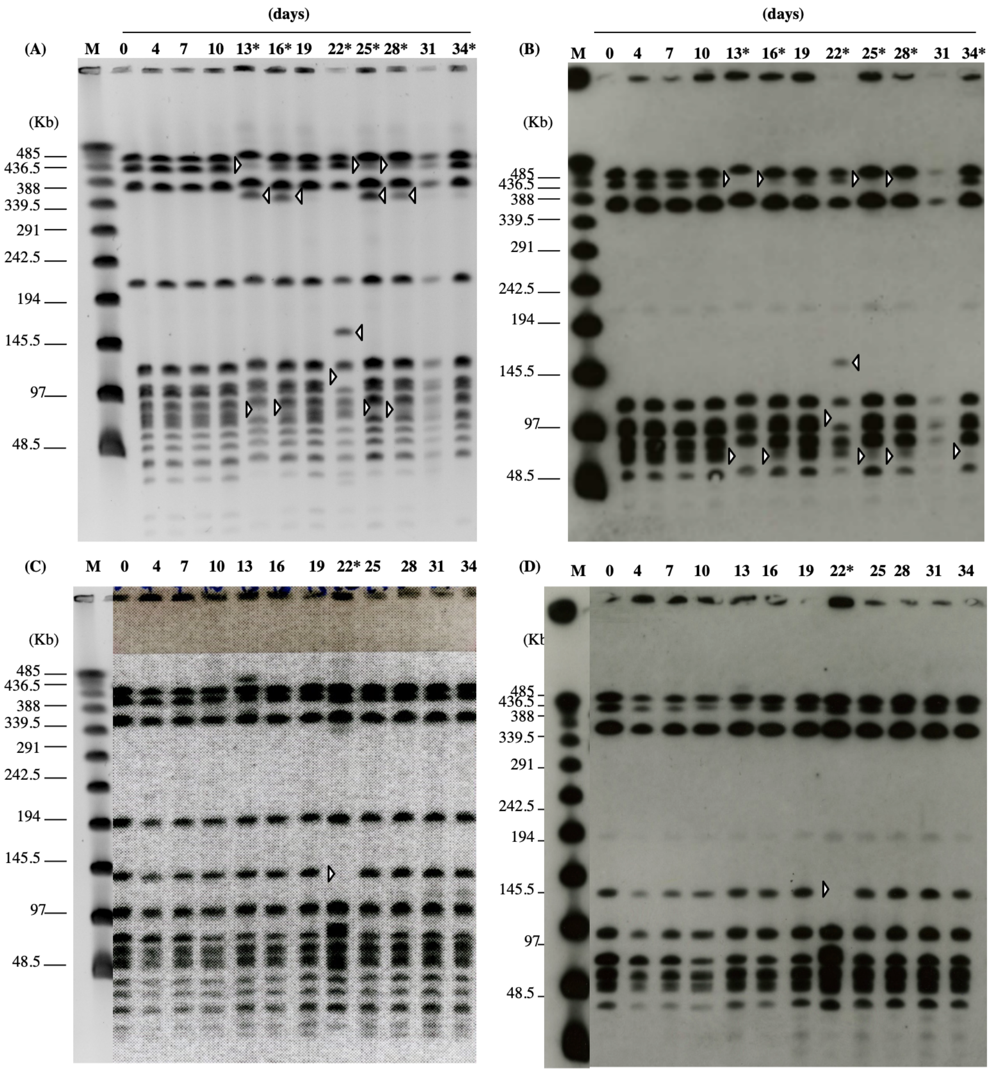
(A)
(B)
(C)
(D)
(days)
M 0 4 7 10 13* 16* 19 22* 25* 28* 31 34*
M 0 4 7 10 13 16 19 22* 25 28 31 34
(Kb)
485
436.5
388
339.5
291
242.5
194
145.5
97
48.5

FIG S1 In vitro stability assays. (A) In vitro stability over time of SmaI PFGE profiles. (B) In vitro stability over time of IS1272- hybridization patterns. (C) In vitro stability over time of SmaI PFGE profiles in the presence of oxacillin. (D) In vitro stability over time of IS1272- hybridization patterns in the presence of oxacillin. Lane M: λ ladder used as a size marker. The positions of molecular markers (in kilobase pairs) are indicated by black lines. The white arrows indicate band loss/gain in PFGE and SmaI-IS1272 profiles. The arrowheads on the right of each lane correspond to the appearance of a band. The arrowheads on the left of each lane correspond to the disappearance of a band. At the top of each lane the day that the methicillin resistant S. haemolyticus (MRSHae) HSM742 isolate was collected is indicated in relation to the first isolate (day 0). The asterisks indicate days that showed alteration.

Figure S2

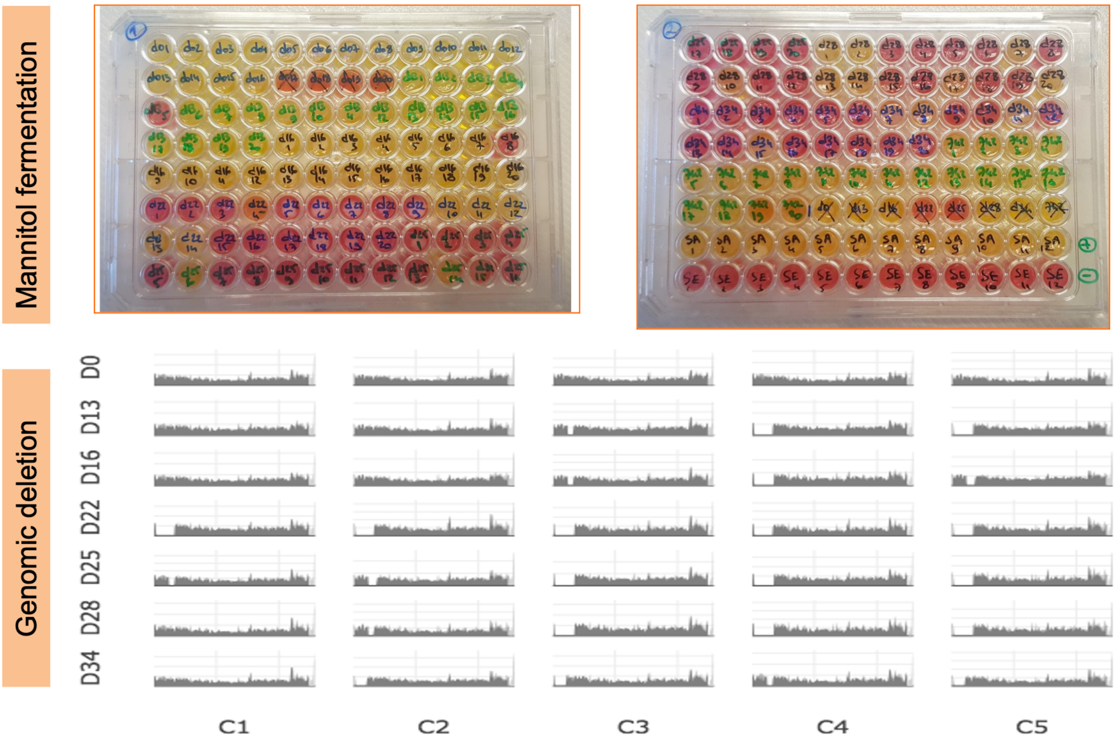

Mannitol +	Mannitol -
20	0
19	1
19	1
5	15
2	18
7	13
3	17

FIG S2 Mannitol fermentation of 20 colonies of HSM742 variants in seven time points of the stability assay.

Figure S3

A

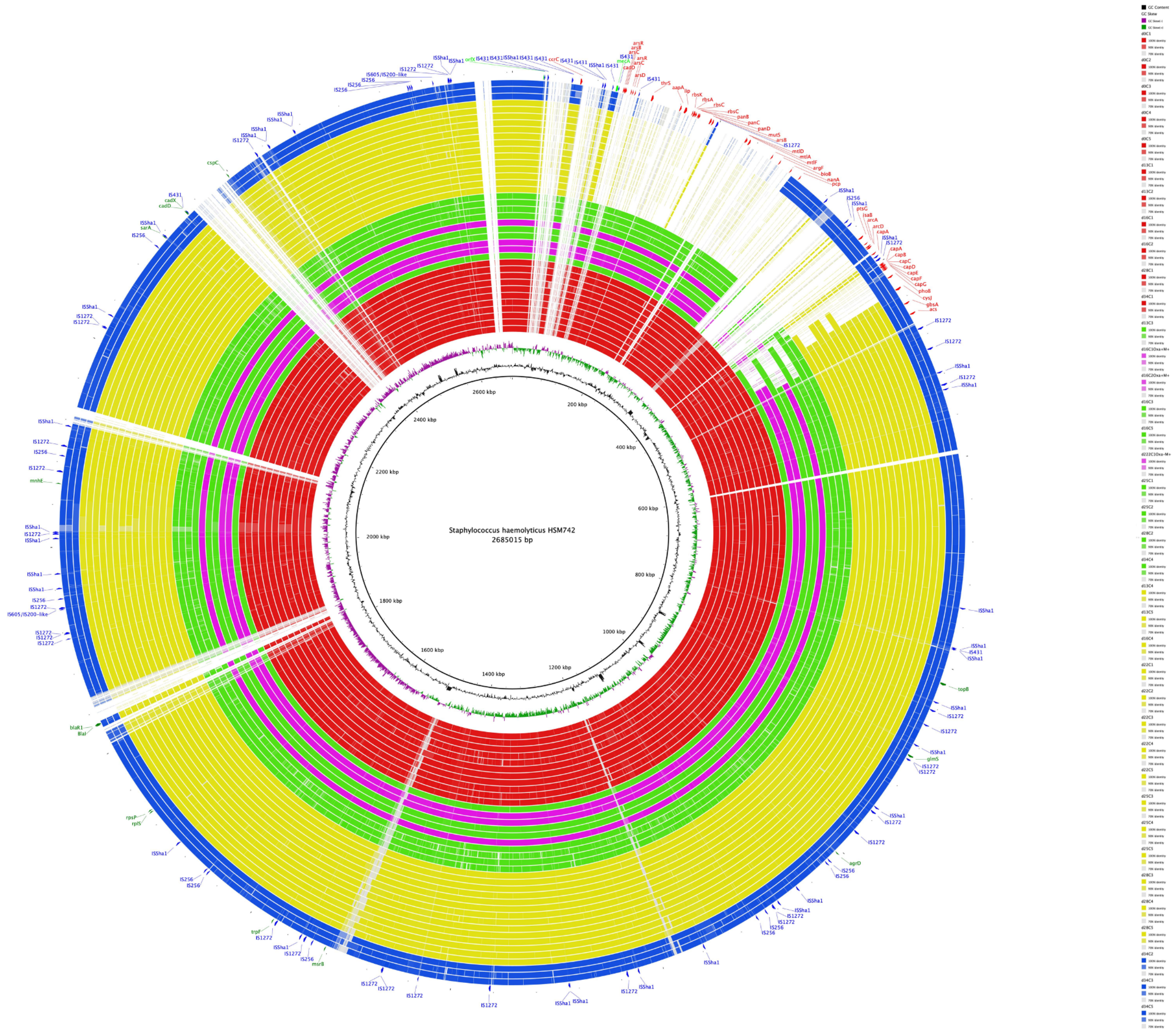

Staphylococcus haemolyticus HSM742
2685015 bp
200 kbp
400 kbp
600 kbp
800 kbp
1000 kbp
1200 kbp
1400 kbp
1600 kbp
1800 kbp
2000 kbp
2200 kbp
2400 kbp
2600 kbp
topB
agrD
mnhE
blaR1
blaI
trpF
msrB
cspC

B

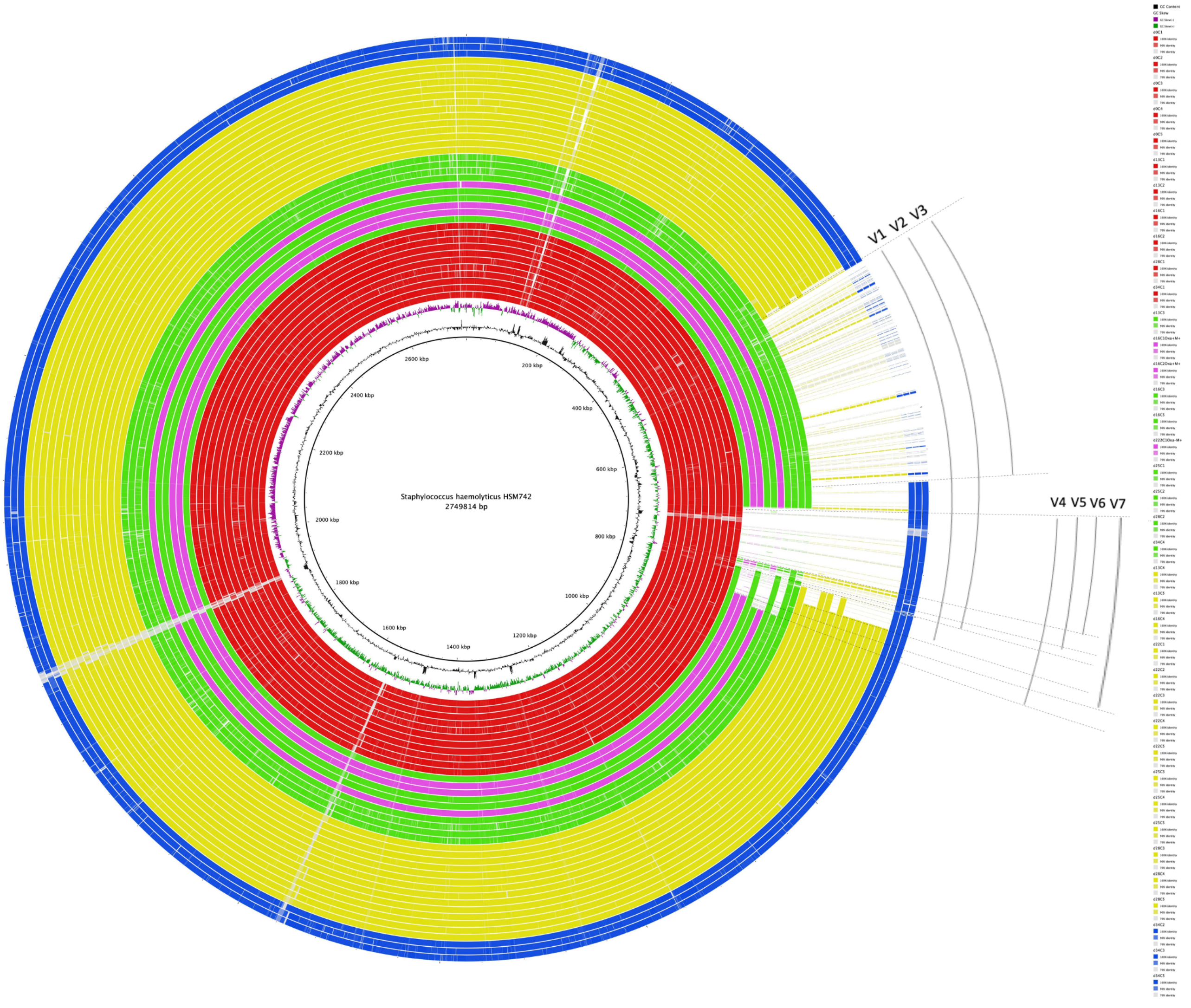
Staphylococcus haemolyticus HSM742
2749814 bp
200 kbp
400 kbp
600 kbp
800 kbp
1000 kbp
1200 kbp
1400 kbp
1600 kbp
1800 kbp
2000 kbp
2200 kbp
2400 kbp
2600 kbp
V1 V2 V3
V4 V5 V6 V7

FIG S3 Blast Ring Image Generator (BRIG) diagram showing homologous chromosome segments of S. haemolyticus HSM742 variants V1-V7 with genomes of strain JCSC1435 or HSM742d0 as references.
